# Diagnostic Accuracy of Intraoperative Touch Imprint Cytology for the Diagnosis of Axillary Sentinel Lymph Node Metastasis of Breast Cancer: Comparison With Intraoperative Frozen Section Evaluation

**DOI:** 10.7759/cureus.12960

**Published:** 2021-01-28

**Authors:** Atif A Hashmi, Samreen Naz, Omer Ahmed, Syed Rafay Yaqeen, Anoshia Afzal, Ishaq Azeem Asghar, Muhammad Irfan, Naveen Faridi

**Affiliations:** 1 Pathology, Liaquat National Hospital and Medical College, Karachi, PAK; 2 Internal Medicine, Liaquat National Hospital and Medical College, Karachi, PAK; 3 Internal Medicine, Baqai Medical University, Karachi, PAK; 4 Pathology, University of Oklahoma Health Sciences Center, Oklahoma City, USA; 5 Pathology, Ascension St. John Hospital, Detroit, USA; 6 Statistics, Liaquat National Hospital and Medical College, Karachi, PAK

**Keywords:** frozen section, touch imprint cytology, sentinel lymph nodes, breast cancer

## Abstract

Introduction

The intraoperative frozen section is a recommended method to detect breast cancer metastasis to axillary sentinel lymph nodes (SLNs); however, frozen section is not widely available and requires an experienced staff. Alternatively, touch imprint cytology (TIC) is a simple and cost-effective technique to detect metastasis. Therefore, in this study, we assessed the diagnostic accuracy of TIC for detecting SLN metastasis and compared it with intraoperative frozen section evaluation.

Methodology

A retrospective study was conducted in the Department of Histopathology, Liaquat National Hospital and Medical College, for a duration of two years. A total of 114 patients undergoing surgery for primary breast cancer were included in the study. All patients had clinically and radiologically negative axillary lymph nodes. SLN sampling was done using radioactive dye and sent for intraoperative consultation. The SLNs were sliced at 4-mm intervals and two TIC slides and three step-levels for frozen section were prepared, and the results were compared with final (paraffin) section histology.

Results

The sensitivity, specificity, and diagnostic accuracy of TIC was 83.7%, 98.5%, and 92.1%, respectively. Alternatively, the sensitivity, specificity, and diagnostic accuracy of frozen section was 93.9%, 100%, and 97.4%, respectively. The sensitivity of TIC and frozen section for detecting micrometastasis was 14.3% and 57.1%, respectively, with a diagnostic accuracy of 90.3% and 95.8%, respectively. Alternatively, with respect to macrometastasis, the sensitivity and specificity of TIC were 95.2% and 98.5%, respectively, while the sensitivity and specificity of frozen section were 100%.

Conclusion

TIC is a quick and effective technique for detecting breast cancer metastasis in axillary SLNs. Although frozen section had an overall higher sensitivity than TIC, the sensitivity of TIC for detecting macrometastasis was comparable to the frozen section. Therefore, we conclude that TIC is a good alternative to the frozen section in facilities where the frozen section is not available.

## Introduction

Breast cancer is a prevalent cancer in women worldwide. In Pakistan, besides its high prevalence, young age at presentation and poor overall prognosis is especially concerning [1,2]. Metastasis to axillary lymph nodes (LNs) is an important prognostic factor in breast cancer. Alternatively, axillary dissection leads to high morbidity owing to arm edema after surgery. Therefore, a guarded approach is recommended for axillary dissection. For patients with clinically and radiologically positive axillary LNs, axillary LN dissection is recommended after fine-needle aspiration (FNA) or core-needle biopsy confirmation of axillary metastasis. Alternatively, in patients with clinically and radiologically negative axillary LNs, sentinel lymph node (SLN) sampling is done. The presence of macrometastasis on SLN examination in one or more SLNs (one SLN in cases with mastectomy and more than three SLNs in cases with breast conservation surgeries) is followed by axillary dissection. To prevent two surgeries, intraoperative frozen section evaluation of SLN is becoming a routine practice in breast cancer surgeries. Frozen section evaluation of SLN has high sensitivity and specificity [3]. However, in resource-limited countries like Pakistan, frozen section facility is not available in most institutions, and so patients have to undergo two surgeries (first for SLN sampling and second for definitive breast cancer surgery). The intraoperative touch imprint cytology (TIC) is another simple and cost-effective technique by which SLN metastasis can be detected [4]; however, its utility is not widely evaluated in our setup. Therefore, in this study, we assessed the diagnostic accuracy of TIC for detecting SLN metastasis and compared it with intraoperative frozen section evaluation of SLNs.

## Materials and methods

**Figure 1:**
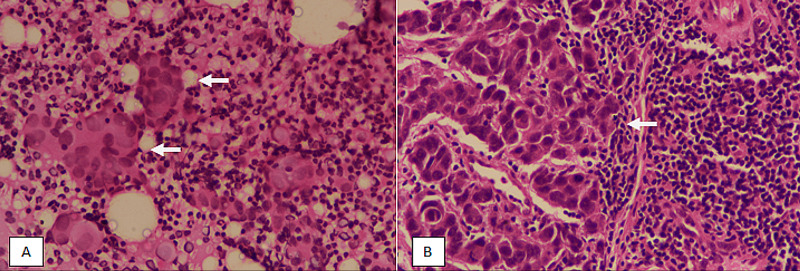
TIC and frozen section. (A): TIC (H&E staining at 400× magnification) showing clusters of large atypical epithelial cells (arrows) consistent with metastatic carcinoma. (B): Frozen section (H&E staining at 400× magnification) showing a deposit of metastatic carcinoma (arrow). TIC, touch imprint cytology; H&E, hematoxylin and eosin

A retrospective study was conducted in the Department of Histopathology, Liaquat National Hospital and Medical College, for a duration of two years. A total of 114 patients undergoing surgery for primary breast cancer were included in the study. Patients with evidence of systemic metastases along with those who received post-neoadjuvant chemoradiation before surgery were excluded from the study. All patients had clinically and radiologically negative axillary LNs. SLN sampling was done using radioactive dye and sent for intraoperative consultation. The number of SLNs was recorded along with size. SLNs measuring smaller than 4 mm were bivalved along the hilum. SLNs measuring larger than 4 mm were bread-loafed at 2 mm interval. Two TIC slides were prepared from each cut surface of all SLNs and stained with DiffQuik and hematoxylin and eosin (H&E) stains. All slices of SLNs were then submitted entirely for frozen section analysis. Three step-levels were prepared from each block and stained with H&E and examined by experienced histopathologists (Figure 1).

After reporting of frozen sections, the remaining tissue from blocks was fixed in formalin and examined by final (paraffin) sections by pathologists. Cytokeratin immunostains were performed on paraffin blocks where necessary. Results of TIC and frozen sections were compared with final (paraffin) histology for detecting metastatic carcinoma.

After reporting SLNs intraoperatively, definite surgery (lumpectomy or mastectomy with or without axillary dissection) was done and the specimen was sent for histopathological examination. Gross examination of the specimen was performed and representative sections were submitted from the tumor, resection margins, normal breast tissue, and axillary lymph nodes and examined by histopathologists.

Data analysis was performed using Statistical Package for Social Sciences (Version 26.0, IBM Inc., Armonk, NY, USA). Sensitivity, specificity, positive predictive value (PPV), negative predictive value (NPV), and diagnostic accuracy were calculated for TIC and frozen sections by 2 × 2 tables using final (paraffin) sections as the gold standard.

## Results

The mean age of the patients was 53.41 ± 12.46 years. Most patients (83.3%) had invasive ductal carcinoma, and the most common histological grade was III (50.9%). A total of 64% of the patients had tumor (T)-stage T2 and 43% had nodal metastasis. Out of the 49 cases of positive SLNs on final (paraffin) histology, 42 (85.7%) had macrometastasis, while 7 (14.3%) had micrometastasis (Table 1).

**Table 1:** Clinicopathological features of the population under study *Mean ± SD (standard deviation)/range SLN, sentinel lymph node; TIC, touch imprint cytology

Clinicopathological features	Frequency (%)
Age (years)*	53.41 ± 12.46/25–83
Histological diagnosis	
Ductal carcinoma	95(83.3)
Mucinous carcinoma	3(2.6)
Lobular carcinoma	4(3.5)
Ductal carcinoma in situ	6(5.3)
Mucinous carcinoma	2(1.8)
Metaplastic carcinoma	2(1.8)
Solid papillary carcinoma	2(1.8)
Histological grade	
Grade I	16(14)
Grade II	40(35.1)
Grade III	58(50.9)
Tumor (T)-stage	
Tis (in situ)	6(5.3)
T1	24(21.1)
T2	73(64)
T3	11(9.6)
Nodal (N)-stage	
N0	65(57.0)
N1	42(36.8)
N2	7(6.2)
Specimen type	
Mastectomy	85(74.6)
Lumpectomy	29(25.4)
Laterality	
Right	67(58.8)
Left	47(41.2)
Total number of SLNs*	5.54 ± 2.68/1–14
Number of positive SLNs (n = 49)*	1.76 ± 1.10/1–5
SLN metastasis size (cm) (n = 49)*	1.05 ± 0.88/0.1–4.0
SLN status	
Micrometastasis	7(6.1)
Macrometastasis	42(36.8)
Negative for metastasis	65(57)
TIC diagnosis	
Positive for metastatic carcinoma	42(36.8)
Negative for metastatic carcinoma	72(63.2)
Frozen section diagnosis	
Positive for metastatic carcinoma	46(40.4)
Negative for metastatic carcinoma	68(59.6)
Final (paraffin) section diagnosis	
Positive for metastatic carcinoma	49(43)
Negative for metastatic carcinoma	65(57)

Table 2 shows the comparison of TIC and frozen section diagnosis with final (paraffin) diagnosis. The sensitivity, specificity, and diagnostic accuracy of TIC was 83.7%, 98.5%, and 92.1%, respectively. Alternatively, the sensitivity, specificity, and diagnostic accuracy of frozen section was 93.9%, 100%, and 97.4%, respectively.

**Table 2:** Comparison of TIC and frozen section diagnosis with final (paraffin) section diagnosis for the detection of SLN metastasis (n = 114). PPV, positive predictive value; NPV, negative predictive value; TIC, touch imprint cytology; SLN, sentinel lymph node

Diagnostic technique	Diagnosis	Final (paraffin) section diagnosis			Sensitivity, specificity, PPV, NPV, diagnostic accuracy
		Positive	Negative	Total	
TIC diagnosis	Positive	41	1	42	83.7%, 98.5%, 97.6%, 88.9%, 92.1%
	Negative	8	64	72	
	Total	49	65	114	
Frozen section diagnosis	Positive	46	0	46	93.9%, 100%, 100%, 95.6%, 97.4%
	Negative	3	65	68	
	Total	49	65	114	

The sensitivity of TIC and frozen section for detecting micrometastasis was 14.3% and 57.1%, respectively, with a diagnostic accuracy of 90.3% and 95.8%, respectively (Table 3).

**Table 3:** Comparison of TIC and frozen section diagnosis with final (paraffin) section diagnosis for the detection of SLN micrometastasis (n = 72). PPV, positive predictive value; NPV, negative predictive value; TIC, touch imprint cytology; SLN, sentinel lymph node

Diagnostic technique	Diagnosis	Final (paraffin) section diagnosis			Sensitivity, specificity, PPV, NPV, diagnostic accuracy
		Positive (micrometastasis)	Negative	Total	
TIC diagnosis	Positive	1	1	2	14.3%, 98.5%, 50%, 91.4%, 90.3%
	Negative	6	64	70	
	Total	7	65	72	
Frozen section diagnosis	Positive	4	0	4	57.1%, 100%, 100%, 95.6%, 95.8%
	Negative	3	65	68	
	Total	7	65	72	

Alternatively, with respect to macrometastasis, the sensitivity and specificity of TIC was 95.2% and 98.5%, respectively, while the sensitivity and specificity of frozen section were 100% (Table 4).

**Table 4:** Comparison of TIC and frozen section diagnosis with final (paraffin) section diagnosis for the detection of SLN macrometastasis (n = 107). PPV, positive predictive value; NPV, negative predictive value, TIC, touch imprint cytology; SLN, sentinel lymph node

Diagnostic technique	Diagnosis	Final (paraffin) section diagnosis			Sensitivity, specificity, PPV, NPV, diagnostic accuracy
		Positive (macrometastasis)	Negative	Total	
TIC diagnosis	Positive	40	1	41	95.2%, 98.5%, 97.6%, 97%, 97.2%
	Negative	2	64	64	
	Total	42	65	107	
Frozen section diagnosis	Positive	42	0	42	100%, 100%, 100%, 100%, 100%
	Negative	0	65	65	
	Total	42	65	107	

## Discussion

In this study, we compared the performance of TIC and frozen section for detecting breast cancer metastasis to axillary SLNs. While the diagnostic accuracy of the frozen section is higher compared to TIC, the sensitivity of TIC for the detection of macrometastasis is comparable to the frozen section. According to current guidelines, although micrometastasis in axillary SLNs is considered at N1 stage, axillary dissection is not recommended, which makes TIC a useful technique for SLN evaluation as its sensitivity was found to be good for the detection of macrometastasis. Moreover, TIC has the advantage of rapid results and low cost; alternatively, frozen section assessment requires experienced and trained technologists and pathologists.

TIC is recommended for use in resource-limited areas as a first step intraoperatively, followed by a frozen section, if needed/applicable to the situation [5]. Moreover, there are a few more advantages of TIC over frozen section in addition to being cost-effective and sensitive. TIC does not waste any tissue and a pathologist/histotechnologist does not have to deal with fatty lymph nodes and the impedance the fat causes during tissue cutting along with the freezing artifact with TIC [6]. Despite these advantages of TIC over frozen section, TIC has some limitations. The major disadvantage of TIC is that the differentiation of macrometastasis from micrometastasis is not possible with TIC. As the axillary dissection is recommended only for macrometastasis, this limitation has major implications on surgical management and should be communicated to the operating surgeon. Conversely, the sensitivity of TIC for detecting micrometastasis is also low and gross examination of SLNs can help resolve this issue in a few cases. Macrometastatic disease detection with the TIC is almost the same as frozen section, and with proper technique and experience, even micrometastasis detection/sensitivity can improve [5,6]. Accurate assessment requires experience and proper technique usage. There is no consensus with respect to TIC for optimal evaluation of axillary SLNs intraoperatively, but studies have shown TIC to be a reliable and sensitive tool where the frozen section facility is not available [7-10].

Previous studies have estimated the sensitivity of TIC ranging 29-94%, accuracy ranging 78-98%, and specificity ranging 88-100% [4-7,11]. We found the sensitivity of TIC to be 83.7%, with a specificity of 98.5%. Studies have shown that the combined frozen section and TIC were as effective as frozen section or TIC alone [4,7,12]. However, metastatic carcinomas and lobular breast carcinoma detection were less accurate with TIC.

Several studies have proven the effectiveness of TIC for detecting macrometastasis and have recommended it during most surgeries followed by frozen section evaluation if needed or if the TIC is negative and clinical concern is high [5-7,13]. Pétursson et al. found TIC to be highly specific and moderately sensitive for the detection of breast cancer, and interestingly, the sensitivity improved over years, which suggests that implementing TIC in routine settings will increase the overall detection rates and decrease false-negative results over time. The study also concluded that there were no differences in the sensitivity of TIC for different subtypes of breast cancer [14].

Some studies have suggested adding intraoperative cytokeratin immunohistochemistry to decrease false-negative rates with TIC [13]; however, resource-limited settings may not be able to implement these suggestions.

We acknowledge a few limitations of our study. As it was a single center study with a relatively small sample size, we recommend large-scale, multi-center studies to further uncover the diagnostic utility of intraoperative TIC for detecting breast cancer metastasis to axillary SLNs.

## Conclusions

Although sensitivity of frozen section was better than TIC for detecting breast cancer metastasis to axillary SLNs, TIC has several advantages over frozen section, such as low cost and rapid turnover time. We also found that TIC had a high sensitivity and specificity for detecting macrometastasis, which makes TIC a useful method to detect breast cancer metastasis to SLNs in institutions where the frozen section facility is not available. However, the differentiation of macrometastasis from micrometastasis is not possible by TIC; therefore, careful gross examination of SLNs is of utmost importance in the absence of frozen section evaluation.
